# Spontaneous Rupture of Rhabdomyosarcoma of the Testis With Unilateral Ptosis: A Case Report and Literature Review

**DOI:** 10.3389/fped.2022.904275

**Published:** 2022-06-28

**Authors:** Ronghua Wu, Xing Liu, Yajun Song, Shanhong Yi, Wei Chen, Wanlei Fu, Jingzhen Zhu

**Affiliations:** ^1^Department of Urology, Xinqiao Hospital of Army Medical University, Chongqing, China; ^2^Department of Pathology, Xinqiao Hospital of Army Medical University, Chongqing, China

**Keywords:** spontaneous rupture, rhabdomyosarcoma, testis, unilateral ptosis, case report

## Abstract

Spontaneous rupture of testicular rhabdomyosarcoma is very rare. We report a case of spontaneous testicular rupture that was pathologically confirmed as rhabdomyosarcoma with unilateral blepharoptosis. The patient, a 19-year-old male, and his father had weakness of the left eyelid muscle. The patient was suspected to have a right inguinal hernia by a family doctor but was not treated further. 2 days later, there was skin itching in the right inguinal area, accompanied by redness, swelling and discomfort of the right scrotum, and the patient went to the local hospital again. Ultrasound examination showed that a contusion of the right testis may have been complicated with orchitis. Oral levofloxacin was ineffective. In addition, the swelling of scrotal increased significantly. He came to the emergency room of our hospital and also was treated with levofloxacin, but the pain was still not relieved. CT and ultrasound examination could not identify the cause of the disease. Exploration of the right scrotum was performed under general anesthesia and confirmed that the right testis had spontaneously ruptured. The pathological diagnosis was rhabdomyosarcoma of the right testis. Testicular rhabdomyosarcoma is clinically rare, and spontaneous rupture is even rarer. The pathogenesis of the disease needs to be further studied, and the diagnosis should be made on a case-by-case basis. Overall, the prognosis of testicular rhabdomyosarcoma is poor. As seen in this case, further study is required to determine whether there is some association between testicular rhabdomyosarcoma and ptosis. Unfortunately, the patient's family rejected a genetic examination because of financial difficulty. We only report a single case of this rare phenomenon here.

## Background

Testicular tumors constitute <1% of all male cancers and occur especially between the ages of 15 and 35 years. Testicular germ cell tumor is the most common type of testicular malignancies (approximately 95% of all testicular tumors). The remaining (5%) involves non-germ cell tumors such as sex-cord stromal tumors, testicular lymphomas, and paratesticular tumors [sarcoma (liposarcoma, leiomyosarcoma, rhabdomyosarcoma)] (1). As a non-germ cell tumor, rhabdomyo-sarcoma of the testis is relatively rare in the clinic (2). It is often found by self-examination as the main complaint, and some patients may be comorbid with orchitis or epididymitis (3). Clinically, it is difficult to diagnose the disease before surgery, and postoperative pathology is needed for confirmation.

## Case Presentation

**Figure 1:**
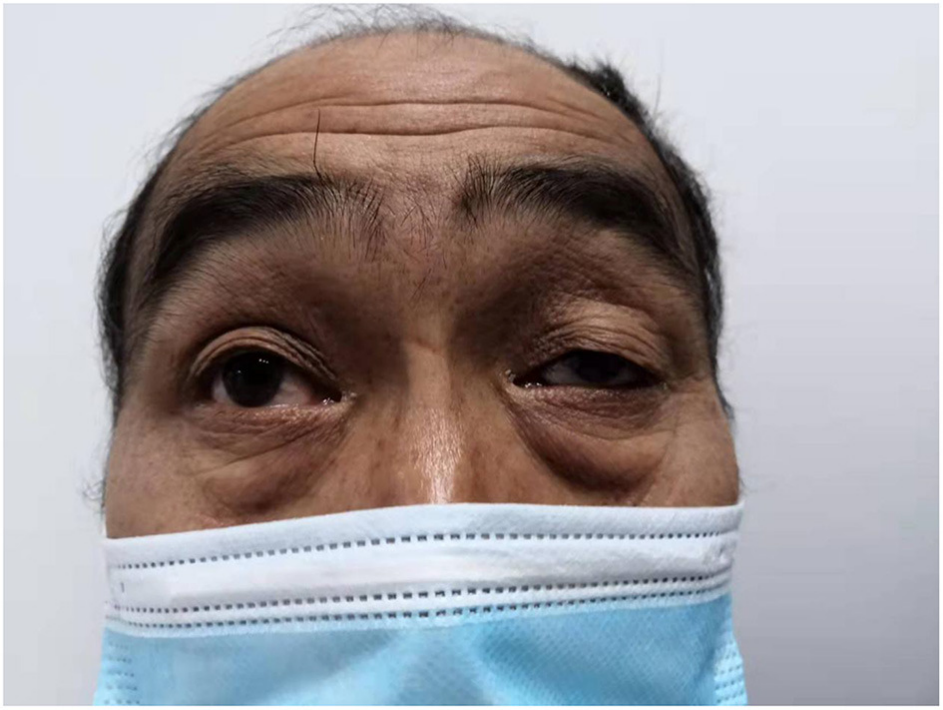
Father had weakness of the left eyelid muscle.

**Figure 2:**
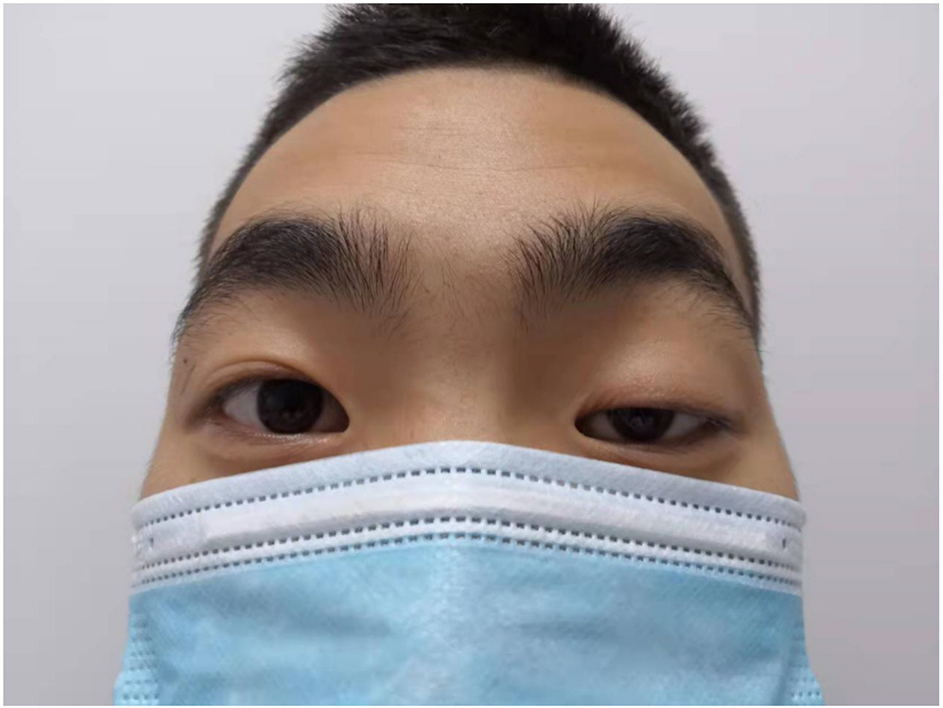
Patient had weakness of the left eyelid muscle.

**Figure 3:**
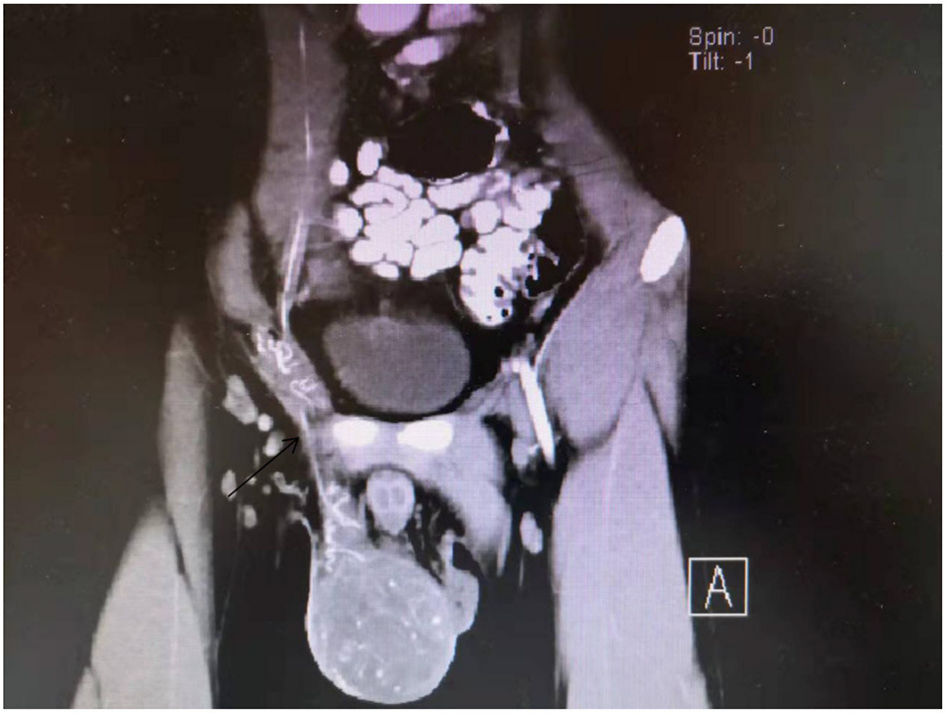
Enhanced CT scan show that the spermatic vessels.

Both the patient, a 19-year-old male who was previously healthy, and his father had weakness of the left eyelid muscle (Figures 1, 2), although their vision was normal. The patient's mother and sister were in good health. After lifting heavy objects, the patient presented with intermittent distending pain and discomfort in the right inguinal region, he did not pay attention to these symptoms and did not seek medical treatment. As the patient's symptoms worsened, a right inguinal hernia was suspected and diagnosed by the family doctor but was not treated further. 2 days later, there was skin itching in the right inguinal area, accompanied by redness, swelling and discomfort of the right scrotum, and he went to the local hospital again. Ultrasound examination showed that a contusion of the right testis may have been complicated with orchitis (no specific medical data). Rest was recommended. The pain was not relieved by oral levofloxacin but resolved on its own 3 days later; nevertheless, it became aggravated and unbearable after exercise. At the same time, the scrotal swelling had significantly increased. He came to the emergency room of our hospital, where a plain CT scan showed scrotal enlargement of unknown cause. Upon admission, the right scrotum was obviously enlarged and tender. It had a high surface temperature, normal skin color, negative scrotal elevation test, negative light transmission test, normal sex hormones and normal tumor markers (including Alpha-fetoprotein, chorionic gonadotropin, carbohydrate antigen-199, carbohydrate antigen-125, serum ferritin, and carcinoembryonic antigen). After admission, the patient was treated with levofloxacin, but the pain was still not relieved. On the second day after admission, ultrasound showed heterogenous echo of the right testis. Considering the possibility of inflammatory lesions, neither testicular tuberculosis nor a tumor could be ruled out. A plain + enhanced abdominal CT scan suggested that inflammatory lesions of the testis with necrosis were possible. In addition, there were some secondary changes in the spermatic vessels, and the spermatic vessels could be seen more clearly (Figure 3). After communicating with the patient and his family, he gave informed consent for exploration of the right scrotum, which was performed under general anesthesia.

## Operation Procedure

**Figure 4:**
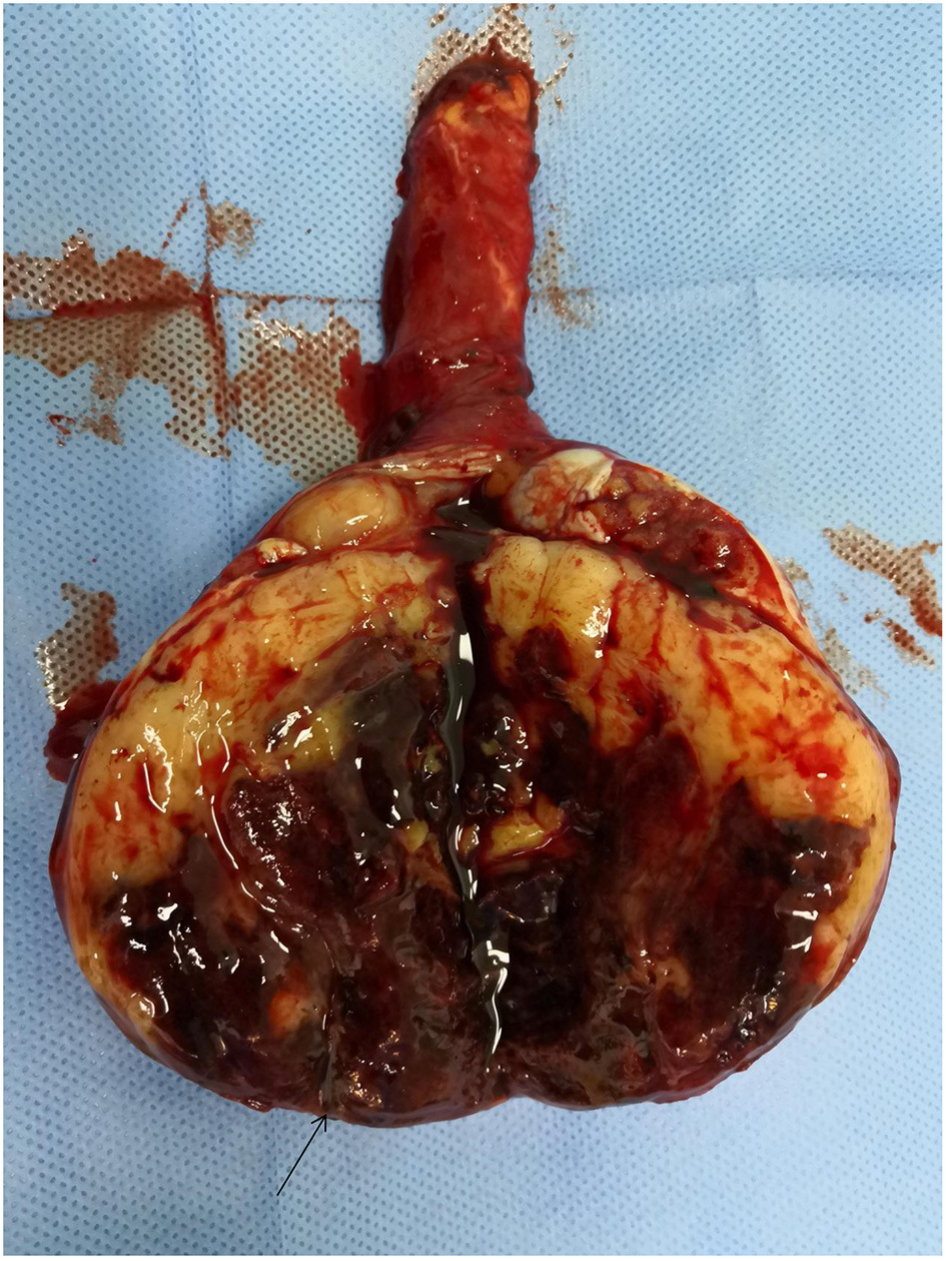
Surgical specimen: the rupture of testicular tunica albuginea.

We made a right groin incision and cut the skin subcutaneously and each layer of muscle in turn, exposing the spermatic cord. We observed hyperaemia and oedema of the spermatic cord blood vessels and surrounding tissue. We rotated the testis approximately 90 degrees, lifted the testis out of the scrotum, opened the testicular sheath, and observed a small amount of bloody fluid within. We opened the tunica albuginea, where we observed a large amount of bloody fluid and a fish-like tissue bulge. The whole testicular boundary was unclear, the epididymis was enlarged, and the tunica albuginea had a visible tear of approximately 1.5 cm in length (Figure 4). Radical orchiectomy was performed on the right side, the spermatic cord was resected in a high position, and the incision was sutured step by step.

## Postoperative Pathology

**Figure 5:**
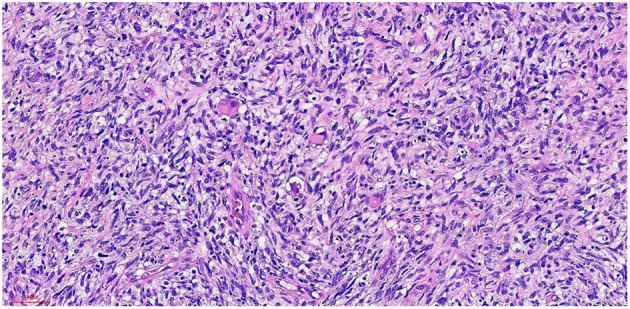
He x 20: The tumor cells were arranged in fascicles, and some were nestled in some areas. The nucleus was fusiform and oval, and the cytoplasm was rich and acidophilic.

**Figure 6:**
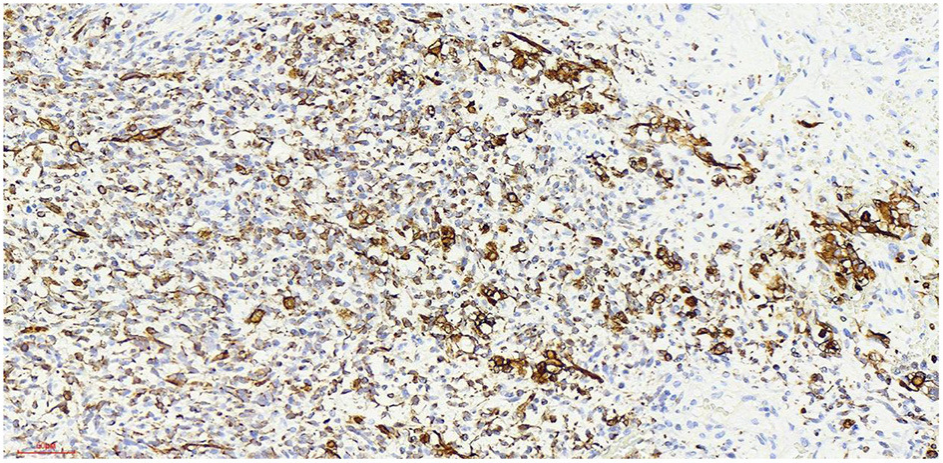
Desmin x 20, +, more cytoplasmic staining of Desmin-positive tumor cells can be seen.

**Figure 7:**
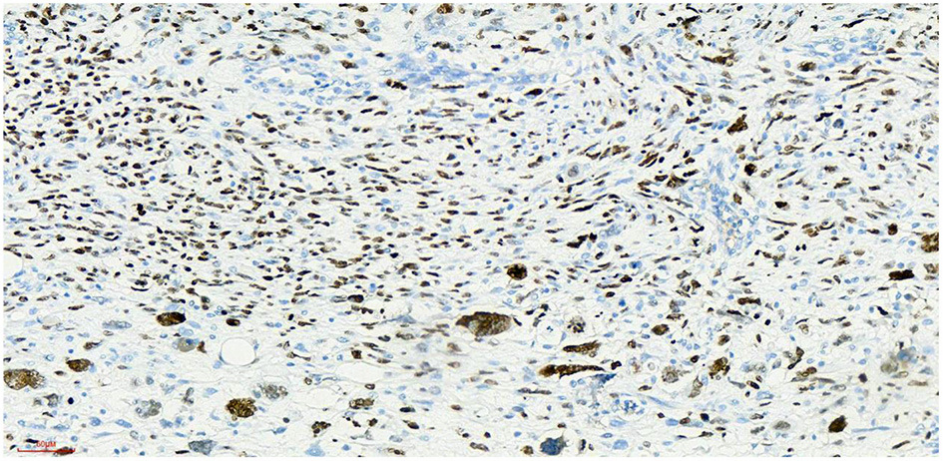
Myod1 x 20, +, nucleoprotein staining showed diffuse expression of Myod1-positive tumor cells.

**Figure 8:**
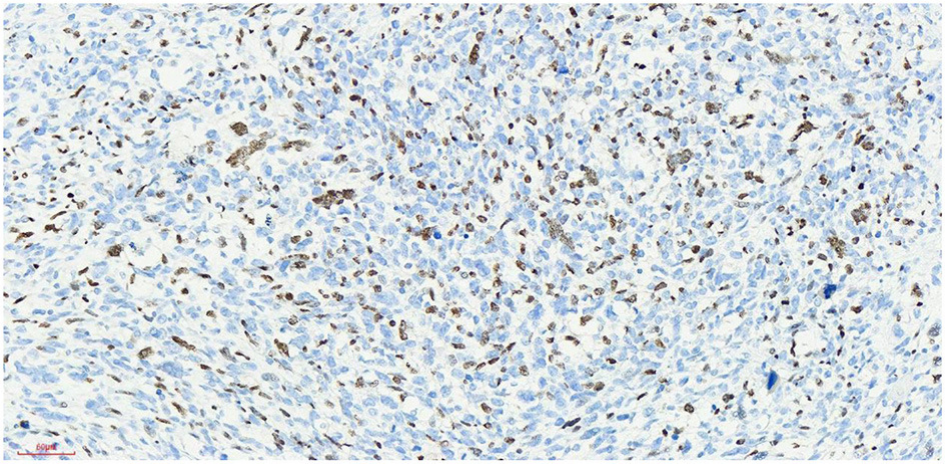
Myogenin x 20, +, visible nuclear protein staining Myogenin positive tumor cells partially expressed.

**Figure 9:**
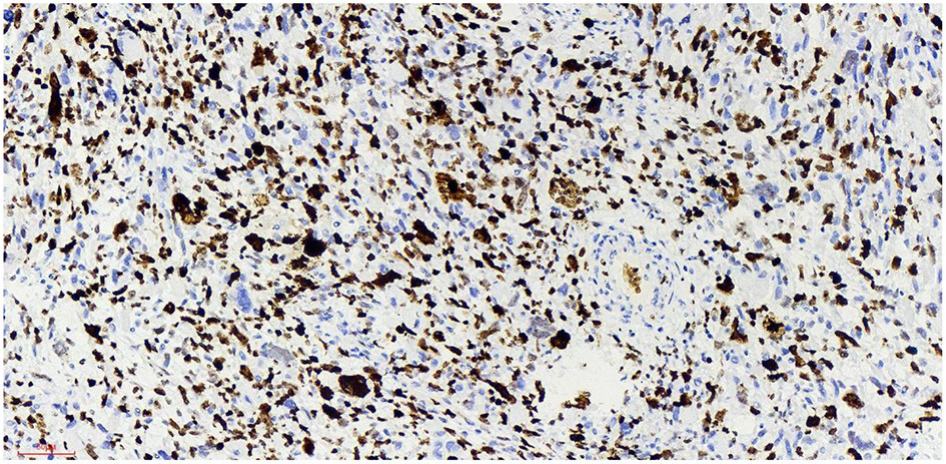
Ki-67 x 20, +, visible nuclear protein staining Ki-67 positive tumor cells diffuse expression, positive rate of 80%.

Haematoxylin-eosin staining (Figure 5). Immunohistochemistry results: Desmin (partly +, Figure 6), SMA (-), MyoD1 (+, Figure 7), Myogenin (+, Figure 8), CD34 (1), STAT-6 (1), S-100 (-), SOX-10 (-), CK (-), EMA (-), TLE1 (-), Ki-67 (80%+, Figure 9). The pathological diagnosis was rhabdomyosarco-ma of the right testis. The postoperative chromosome karyotype analysis showed that there was no Y chromosome microdeletion. Positron emission tomography (PET) examination showed no systemic metabolic abnormalities and no systemic metastasis. These findings suggested that the patient should be treated with a VAC regimen (vincristine, doxorubicin, cyclophosphamide). The patient refused further systemic treatment for personal reasons and is currently under follow-up.

## Discussion and Conclusion

Rhabdomyosarcoma is a very rare malignant tumor derived from striated muscle cells and mesenchymal cells that have differentiated into rhabdomyosarcoma (4). For suspected testicular tumors, preoperative routine testicular biopsy is not recommended because it may cause tumor spread, its incidence is low, accounting for approximately 3% of adult soft tissue tumors (5). It often occurs in the trunk, limbs and other parts of the body. Approximately 20% of urinary system rhabdomyosarcomas occur in the testis or epididymis (6). According to the tissue source, testicular tumors can be divided into reproductive testicular tumors and non-reproductive testicular tumors. Primary rhabdomyosarcoma in the testis and adjacent testes is a rare non-reproductive tumor and the third most common soft tissue sarcoma. In 2002 (7), the WHO classified testicular rhabdomyosarcoma into three types: embryonic testicular rhabdomyosarcoma (fusiform cell rhabdomyosarcoma, grape cluster rhabdomyosarcoma, anaplastic rhabdomyosarcoma), acinar rhabdomyosarcoma and pleomorphic rhabdomyosarcoma.

### Established Facts

At present, the etiology of testicular rhabdomyosarcoma is unknown. Some scholars believe that it may be related to the excessive differentiation and growth of undifferentiated mesenchymal cells in the testis or primordial germ cells in a testicular teratoma (8). Other causes include cryptorchidism and the use of exogenous hormones. Mutation of the P16 gene and abnormal expression of the Rb protein may be related to the occurrence and development of testicular rhabdomyosarcoma (9). The patient of this case and his family refused genetic testing, so it was impossible to determine whether the tumorigenesis was caused by gene deletion or mutation. Skrzypek (10) reported that the immunohistochemical markers myocyte regulatory factor, myogenin and desmin were highly specific to rhabdomyosarcoma and were expressed in almost all rhabdomyosarcoma cells. The immunohistochemistry of this case showed that: myod1, myogenin and desmin were all expressed. The expression of Ki-67 reflects the proliferative activity of tumor cells and is closely related to the development, metastasis and deterioration of malignant tumors, but it is not specific to specific tumors. Ki-67 was expression positive rate of 80% in this case,

Ultrasound is the primary examination modality in the diagnosis of testicular tumors, characterized by non-invasiveness and high accuracy. However, rhabdomyosarcoma of the testis lacks specific ultrasound features (11), and it is difficult to distinguish rhabdomyosarcoma from other germ cell tumors of the testis, especially when the disease is complicated with orchitis and epididymitis. It can be characterized by heterogeneous echoes and increased blood supply, which makes it difficult to identify in the early stage. This patient underwent several ultrasonographic examinations, all of which suggested that inflammation should not be excluded. When orchitis and epididymitis are treated with antibiotics, they can be relieved and generally do not show the clinical manifestation of progressive pain aggravation, which is one of the clinical differential diagnoses. CT and MRI also lack specificity in revealing the nature of the tumor; heterogeneous echoes and liquefied necrosis can be seen on MR imaging, and spermatic vessels can be seen on enhanced CT (12), which has led to misjudgement of whether there is torsion of the spermatic cord. Retrospective analysis of the patient's tunica albuginea rupture showed that it was due to testicular congestion and swelling caused by spermatic cord torsion. At present, it is not clear whether spermatic cord torsion occurs again after testicular swelling and albuginea rupture. Right inguinal hernia was diagnosed by the family doctor, and rupture of the tunica albuginea of the testis may have occurred at this time, resulting in persistent aggravation of pain.

Testicular rhabdomyosarcoma has a high degree of malignancy and early metastasis. Postoperative chemotherapy can significantly improve the survival rate of patients (13). Testicular rhabdomyosarcoma is sensitive to chemotherapy, and the traditional commonly used chemotherapy regimens are vincristine, doxorubicin, and cyclophosphamide (VAC) (14). With greater understanding of the testicular rhabdomyosarcoma, doxorubicin or pirarubicin (both with low cardiotoxicity) combined with cyclophosphamide can also be used, and the effective rate of chemotherapy remains high. Routine retroperitoneal lymph node dissection after radical orchiectomy remains controversial. Some scholars believe that imaging examination has a high false-positive and false-negative rate in judging retroperitoneal lymph node metastasis (15). Moreover, complications after lymph node dissection are common, such as limb oedema, ejaculatory disturbance and intestinal obstruction. Chemotherapy can treat lymph node metastasis. The PET examination of this patient was negative, and retroperitoneal lymph node dissection was not recommended.

### Novel Insights

The patient also had a special clinical manifestation, that is, unilateral congenital blepharoptosis, similar to his father. Blepharoptosis is often seen along with weakness of the eye muscles (16). This patient had no history of ocular nerve trauma. Myasthenia is an autoimmune disease mediated by a variety of antibodies, cellular immune dependence and complements. It is usually characterized by asymmetric ptosis and/or binocular diplopia, is more severe in the morning and evening, and fluctuates, and some patients will develop systemic myasthenia gravis in the later stage (17). The patient had no symptoms, such as impaired vision, except ptosis. At present, the most studied treatments for myasthenia gravis include anti-acetylcholine receptor antibody (acetylcholine receptor antibody, AChR-Ab), anti-muscle-specific tyrosine kinase antibody (muscle-specific kinase antibody, MuSK-Ab), anti-LPR4 antibody (LPR4-antibody, LPR4-Ab), complement C5, and the cytokines IL2 and IL6 (18). Thymus CT, head and neck MRI, orbital MRI, single-fiber electromyography, repetitive nerve stimulation and head and neck CTA were all refused by the patient because of their cost. Whether the patient's own testicular rhabdomyosarcoma was related to blepharoptosis, whether there was a common pathogenic factor or signaling pathway, and whether it was related to his family history and other conditions were unknown because the patient refused further study.

Testicular rhabdomyosarcoma is clinically rare, and spontaneous rupture is rarer. Some information about the patient is limited. 3 months after the operation, the patient came to the clinic for follow-up once. It was suggested that the patient should have MDT discussion and gene testing or go to the oncology department. The family members of the patient refused due to economic reasons. Only one retroperitoneal ultrasound examination was performed, which suggested that the suspected lymph node was enlarged. Tumor markers were not be detected after the operation. As seen in this case, further study is needed to determine whether there is some association between testicular rhabdomyosarcoma and ptosis, This is where we are very interested in this case report. Both he and his father have unilateral ptosis. We suspect that this is a genetic manifestation. Whether there is an internal relationship between this genetic manifestation and the spontaneous rupture of testicular rhabdomyosarcoma has not been reported in the literature. It's also possible the relationship between ptosis and testicular rhabdomyosarcoma just a co-occurrence. Regardless of the relationship between them, there is no literature to report these two clinical manifestations at the same time and we will conduct further follow-up of the patient to obtain more disease-related information.

## Data Availability Statement

The original contributions presented in the study are included in the article/supplementary material, further inquiries can be directed to the corresponding authors.

## Ethics Statement

The studies involving human participants were reviewed and approved by Medical Ethics Committee of Second Affilliated Hospital of Army Medical University, PLA. The patients/participants provided their written informed consent to participate in this study. Written informed consent was obtained from the individual(s) for the publication of any potentially identifiable images or data included in this article.

## Author Contributions

RW, YS, SY, and WC were primarily responsible for the surgery. XL contributed to the primary nursing care. WF contributed to the pathological diagnosis. JZ contributed to the design and funding. All authors contributed to the article and approved the submitted version.

## Funding

This study was supported by the National Natural Science Foundation of China (No. 81900690).

## Conflict of Interest

The authors declare that the research was conducted in the absence of any commercial or financial relationships that could be construed as a potential conflict of interest.

## Publisher's Note

All claims expressed in this article are solely those of the authors and do not necessarily represent those of their affiliated organizations, or those of the publisher, the editors and the reviewers. Any product that may be evaluated in this article, or claim that may be made by its manufacturer, is not guaranteed or endorsed by the publisher.

## Abbreviations

CT: computed tomography
MRI: magnetic resonance imaging
AFP: alpha-fetoprotein
HCG: chorionic gonadotropin
CA199: carbohydrate antigen-199
CA125: carbohydrate antigen-125
SF: serum ferritin
CEA: carcinoembr-yonic antigen
WHO: world health organization
CTA: computed tomography angiography
IL6: interleukin-6
IL-8: interleukin-8
SMA: smooth muscle actin
MyoD1: myogenic differentiation 1
CD34(1): cluster of differentiation34(1)
STAT-6(1): signal transducer and activator of transcription 6(1)
SOX-10: sex determining region Y-box-related high motility-box 10
CK: cytokeratin;EMA:epithelial membrane antigen
TLE1: transducin-like enhancer of split 1.

## References

[1] Williamson SR Delahunt B Magi-Galluzzi C Algaba F Egevad L Ulbright TM. The world health organization 2016 classification of testicular germ cell tumours: a review and update from the international society of urological pathology testis consultation panel. Histopathology. (2017) 70:335–46. 10.1111/his.13102 27747907

[2] Bhambhvani HP Greenberg DR Kasman AM Eisenberg ML. Clinicopathologic features, outcomes, and prognostic factors of testicular sarcoma: a population-based study. Int Urol Nephrol. (2021) 53:257–67. 10.1007/s11255-020-02634-4 32895865

[3] Vanderstappen C Denies E Perdaens C Vandendriessche H. Adult testiculair rhabdomyosarcoma, a rare tumour arising in a teratoma with somatic type malignancy: a case report. Urol Case Rep. (2020) 12:101385. 10.1016/j.eucr.2020.101385 33102083 PMC7574148

[4] Ward E DeSantis C Robbins A Kohler B Jemal A. Childhood and adolescent cancer statistics 2014. CA Cancer J Clin. (2014) 64:83–103. 10.3322/caac.21219 24488779

[5] Han T Chen J Luan Y Chen X Yang X Zhang Y. Successful treatment of relapsed testicular embryonal rhabdomyosarcoma with endostar and traditional chemotherapy: a case report. Onco Targets Ther. (2018) 30:5287–91. 10.2147/OTT.S170008 30214234 PMC6124800

[6] Dangle PP Correa A Tennyson L Gayed B Reyes-Múgica M Ost M. Current management of paratesticular rhabdomyosarcoma. Urol Oncol. (2016) 34:84–92. 10.1016/j.urolonc.2015.10.004 26572723

[7] Chen E Ricciotti R Futran N Oda D. Head and neck Rhabdomyosarcoma: clinical and pathologic characterization of seven, *Cases Head Neck Pathol*. (2017) 11:321–326. 10.1007/s12105-016-0771-0 27896667 PMC5550390

[8] Coindre JM. Nouvelle classification de l'OMS des tumeurs des tissus mous et des os [New, W. H. O., classification of tumours of soft tissue and bone]. Ann Pathol. (2012) 32(5. Suppl):S115–6. 10.1016/j.annpat.2012.07.006 23127926

[9] de Lambert G Chargari C Minard-Colin V Haie-Meder C Guérin F Martelli H. Testicular transposition in children undergoing brachytherapy for bladder and/or prostate rhabdomyosarcoma. J Pediatr Surg. (2018) 53:1428–31. 10.1016/j.jpedsurg.2018.04.018 29753523

[10] Skrzypek K Kusienicka A Trzyna E Szewczyk B Ulman A Konieczny P. SNAIL is a key regulator of alveolar rhabdomyosarcoma tumor growth and differentiation through repression of MYF5, and MYOD function. Cell Death Dis. (2018) 9:643. 10.1038/s41419-018-0693-8 29844345 PMC5974324

[11] Yi J Zhou DA Huo JR Wang YH Ma JA. Primary intratesticular rhabdomyosarcoma: a. case report and literature review. Oncol Lett. (2016) 11:1016–20. 10.3892/ol.2015.3987 26893684 PMC4734100

[12] Lin C Donaldson SS Meza JL Anderson JR Lyden ER Brown CK. Effect of radiotherapy techniques (IMRT vs. 3D-CRT) on outcome in patients with intermediate-risk rhabdomyosarcoma enrolled in COG D9803–a report from the Children's Oncology Group. Int J Radiat Oncol Biol Phys. (2012) 82:1764–70. 10.1016/j.ijrobp.2011.01.036 21470795 PMC3154985

[13] Walterhouse D Watson A. Optimal management strategies for rhabdomyosarcoma in children. Paediatr Drugs. (2007) 9:391–400. 10.2165/00148581-200709060-00006 18052409

[14] Mondaini N Palli D Saieva C Nesi G Franchi A Ponchiette R. Clinical characteristics and overall survival in genitourinary sarcomas treated with curative intent: a multicenter study. Eur Urol. (200) 47:468–73. 10.1016/j.eururo.2004.09.013 15774243

[15] Dang ND Dang PT Samuelian J Paulino AC. Lymph node management in patients with paratesticular rhabdomyosarcoma: a population-based analysis. Cancer. (2013) 119:3228–33. 10.1002/cncr.28198 23744806

[16] Sanders DB Wolfe GI Benatar M Evoli A Gilhus NE Illa I. International consensus guidance for management of myasthenia gravis: executive summary. Neurology. (2016) 87:419–25. 10.1212/WNL.0000000000002790 27358333 PMC4977114

[17] Jacob S Viegas S Leite MI Webster R Cossins J Kennett R. Presence and pathogenic relevance of antibodies to clustered acetylcholine receptor in ocular and generalized myasthenia gravis. Arch Neurol. (2012) 69:994–1001. 10.1001/archneurol.2012.437 22689047

[18] Gilhus NE. Myasthenia gravis. N Engl J Med. (2016) 375:2570–81. 10.1056/NEJMra1602678 28029925

